# Preparation with recombinant humanized thyroid-stimulating hormone before radioiodine ablation after thyroidectomy: a systematic review

**DOI:** 10.3747/co.v16i5.306

**Published:** 2009-09

**Authors:** J. Yoo, R. Cosby, A. Driedger

**Affiliations:** * Department of Otolaryngology, Schulich School of Medicine and Dentistry, University of Western Ontario, London, ON; † Cancer Care Ontario, Program in Evidence-Based Care and Department of Clinical Epidemiology and Biostatistics, McMaster University, Hamilton, ON; ‡ Division of Nuclear Medicine, Schulich School of Medicine and Dentistry, University of Western Ontario, London, ON

**Keywords:** Radioiodine ablation, thyroid cancer, recombinant humanized thyroid-stimulating hormone, systematic review

## Abstract

**Background:**

Standard treatment for differentiated thyroid cancer is thyroidectomy followed in selected cases by radioiodine ablation (ra). Recombinant humanized thyroid-stimulating hormone (rhtsh) is an exogenous source of tsh that can be administered to obviate the need for hormone withdrawal. In this systematic review, we analysed the evidence for the therapeutic use of rhtsh for ra preparation.

**Method:**

A systematic review of the medline and embase databases from 1996 through January 2008 selected articles reporting randomized controlled trials, cohort studies, and retrospective studies published in English that compared ra using rhtsh with standard hormone withdrawal.

**Results and Interpretation:**

Stimulation by rhtsh is equivalent to thyroid hormone withdrawal in achieving ablation while avoiding detrimental symptoms of hypothyroidism and significantly lowering the whole-body radiation dose. Furthermore, rhtsh may be the only option for patients who either cannot raise endogenous tsh or who would be at risk from the morbidity of hypothyroidism. Based on the results of validated instruments of physical and mental performance, there is agreement that rhtsh maintains a better quality of life. Studies of cost-effectiveness found that rhtsh-prepared patients lost less time from work and required fewer encounters with health care providers.

## 1. INTRODUCTION

Thyroid cancer is the most common of endocrine malignancy in Canada 1 and the United States 2,3, with the fastest-rising incidence of all cancers. The estimated number of new cases in Canada in 2008 was 4300, and the deaths attributable to thyroid cancer for 2008 were estimated to have been 180 1. In the United States, the incidence is similar 3. Because thyroid cancer is an indolent disease and because it can recur decades after initial diagnosis and definitive primary treatment, people with a thyroid cancer diagnosis require life-long surveillance and follow-up 4.

Patients with differentiated thyroid carcinoma (papillary and follicular) are usually treated surgically, with near-total or total thyroidectomy, frequently followed by adjuvant radioiodine (^131^I) therapy of thyroid remnants 5. Traditionally, patients are prepared for radioiodine ablation (ra) by the withdrawal of thyroid hormone replacement, rendering them hypothyroid. The hypothyroid state increases endogenous levels of thyroid-stimulating hormone (tsh), which stimulates the uptake of ^131^I in any remaining thyroid cells and often also in differentiated thyroid cancer cells 6. The induced temporary hypothyroidism can cause substantial morbidities such as cold intolerance, peri-orbital puffiness, weight gain, constipation, and slow movements, negatively affecting quality of life (qol) 7. In addition, pre-existing multisystem disorders such as cardiac, renal, and psychiatric diseases may be exacerbated during hypothyroidism.

Although the withdrawal time varies, patients are generally off thyroid replacement hormone for 4–6 weeks; they restart within 1 week after ra, with thyroid hormone reaching normal levels within 2 months. Some practitioners initially replace the T4 form of hormone with T3, which is more rapidly metabolized, before a short period of total hormone withdrawal, with the intent of shortening the duration of overt hypothyroidism. However, this protocol has been shown to offer no clear benefit 8.

Since the end of the 1990s, several major changes have occurred in the postsurgical management of differentiated thyroid cancers—for example, appreciation of the importance of thyroglobulin (Tg) assays in follow-up, a parallel appreciation of the relative insensitivity of follow-up ^131^I scans 9, and the concurrent development of recombinant humanized tsh (rhtsh).

Exogenously administered rhtsh avoids thyroid hormone withdrawal and was initially approved for use in patients undergoing periodic ^131^I whole-body scans (wbss) and stimulated Tg measurements during follow-up. With rhtsh, patients can remain on thyroid replacement, thereby avoiding the associated morbidity of hypothyroidism. Administration of rhtsh has proved to be a safe and effective method of stimulating ^131^I uptake in patients being monitored for persistent or recurrent thyroid cancer, while maintaining a euthyroid state 10,11.

In 2006, the American Thyroid Association (ata) published a guideline on the management of patients with differentiated thyroid cancer. The ata taskforce issued a level B recommendation for the use of either thyroid hormone withdrawal or rhtsh for radioiodine remnant ablation 12. Since then, the European Commission and the U.S. Food and Drug Directorate have both approved the use of rhtsh for radioiodine remnant ablation in low-risk post-thyroidectomy patients 13,14.

The efficacy of rhtsh for diagnostic and surveillance purposes has raised the question of its utility in the preparation of patients for ^131^I adjuvant therapy, stimulating a systematic review of the evidence for the use of rhtsh for this purpose. Many countries have already approved a role for rhtsh in therapy of selected low-risk patients, but in some centres, standard practice still limits its use to diagnostic surveillance of previously treated thyroid cancer.

## 2. METHODS

### 2.1 Literature Search Strategy

We searched the medline [January 1996 through January (week 5) 2008] and embase [1996 through February (week 6) 2008] databases for relevant evidence. The search terms used included “thyroid cancer,” “thyroidectomy,” “radioactive iodine ablation,” “thyrotropin,” and “randomized controlled trials.” Relevant articles were selected and reviewed by one reviewer, and the reference lists from those sources were searched for additional trials.

### 2.2 Inclusion Criteria

Articles were selected for inclusion in the systematic review if they were fully published English-language reports, involving human subjects, of randomized controlled trials (rcts), cohort studies, and retrospective studies comparing ra preparation using rhtsh with standard withholding of thyroid hormone therapy in patients with no known metastatic disease. Outcome measures of interest were serum tsh levels, results of post-therapy scans, iodine biokinetics in remnants, serum Tg, urinary iodine excretion, and qol.

## 3. RESULTS

### 3.1 Literature Search

The medline search yielded 323 results, twenty-two of which were potentially relevant and ordered for full review. The embase search yielded 132 results, eight of which were potentially relevant and not already included in the medline search. If several papers were published concerning the same dataset, but with updated results, only the most recent paper was included. Four articles from the search were considered relevant for the therapeutic use of rhtsh as compared with thyroid hormone withdrawal.

Quality of life was reported in only one of the four selected therapeutic studies 15. Because qol was deemed to be an important outcome for the present review, studies evaluating rhtsh for diagnostic monitoring purposes that otherwise met the inclusion criteria for study design and that reported on qol were located and reviewed as well. Consequently, the section reporting qol was supplemented with results from three other studies 11,12,16, all of which compared hypothyroid (hypo) and rhtsh preparation for the diagnostic use of ^131^ I.

### 3.2 Study Characteristics

In all four studies of therapeutic use 15,17–19, 0.9 mg of rhtsh was administered intramuscularly on 2 consecutive days, followed by ra either 24 15,17,19 or 48 18 hours after the second dose of rhtsh. Patients in all four studies had previously undergone total or near-total thyroidectomy. In all of the studies, patients receiving rhtsh continued on thyroxine replacement throughout their treatment. Table I summarizes the studies.

### 3.3 Study Quality

The literature search only yielded one rct 15 on the use of rhtsh therapeutically for ra preparation. Although of good quality, this study enrolled only 63 participants over a period of 21 months in 9 study sites. However, it is important to note that it was this study that led to the approval of rhtsh by the European Medicines Agency. In addition, a large prospective trial on this topic is unlikely, given that the incidence of thyroid cancer is too low for a large trial to be feasible in a reasonable amount of time. Furthermore, the favourable prognosis and protracted nature of thyroid cancer means that it takes many years, and even decades, to reach certain endpoints 4.

The cohort studies by Barbaro *et al.* 17 and Pacini *et al.* 18 and the retrospective review by Robbins *et al.* 19 are limited by lack of randomization, making it unclear whether selection bias (either self-selection by patients or selection by physicians) affected the results of a given study. In addition, Barbaro *et al.* 17 used a historical control group (that is, a thyroid hormone withdrawal group), which is methodologically problematic.

### 3.4 Successful Ablation Rates and Serum Tg Levels

The rct reported by Pacini *et al.* 15 compared ra preparation in 63 patients divided into hypo and euthyroid (rhtsh) arms. In this study, a 3700-MBq dose of ^131^I was used. At 8 months’ follow-up, 86% of the hypo group and 75% of the rhtsh group had a negative wbs and, as a result, were considered successfully ablated (*p* = 0.3). The rate of successful ablation was 100% in both groups when success was defined as no visible uptake or less than 0.1% ^131^I uptake in the thyroid bed. This definition was reasonable, given that low-grade false positive results occur because of salivary pooling in the oropharynx during the scan. Furthermore, 86% and 83% of the hypo and rhtsh groups respectively had serum Tg levels below 1.0 ng/mL (Table II), further indicating that both methods of ra preparation led to successful ablation rates.

The cohort study reported by Barbaro *et al.* 17 compared hypo and rhtsh ra preparation in 93 patients. Notably, patients in the rhtsh group underwent thyroid hormone withdrawal for 4 days: l-T4 was discontinued the day before the first dose of rhtsh and resumed the day after the administration of a 1110- MBq therapeutic dose of ^131^I. The rates of successful ablation were not significantly different between the two groups of patients (*p* value not reported), but at the 1-year follow-up, 75.6% of the hypo group and 76.9% of the rhtsh group had a negative wbs. Serum Tg levels were less than 1.0 ng/mL in 78.0% of the hypo group and in 86.5% of the rhtsh group.

Another cohort study, reported by Pacini *et al.* 18, compared ra preparation in 162 patients. However, in addition to hypo and rhtsh groups, a third group was prepared by both thyroid hormone withdrawal and administration of rhtsh (hypo+rhtsh). A 1110-MBq dose of ^131^I was given to all patients in that series. At 6–10 months’ follow-up, 84% and 78.5% of patients in the hypo and hypo+rhtsh groups respectively had a negative wbs, but only 54% (*p* < 0.0001 and *p* < 0.01) of those in the rhtsh group did. It is noteworthy that, in this study, ^131^I was administered 48 hours after the second rhtsh injection rather than the usual 24 hours, which may have contributed to the lower rate of successful ablation in the rhtsh arm. However, guidelines published by ata 9 indicate that serum Tg is the best indicator for successful ablation. They recommended that the uptake and scan procedure be discontinued. Using this criterion, the successful ablation rates were 88%, 95%, and 74.1% in the hypo, hypo+rhtsh, and rhtsh groups respectively, none of which are significantly different.

Finally, Robbins *et al.* 19 reported retrospectively on a comparison of hypo and rhtsh in 87 patients. This study was unique among those reviewed in that the dosage of ^131^I was individually determined based on dosimetry, rather than administered in a standard fixed dose. For both groups, the mean dose was more than 3700 MBq. Follow-up at approximately 1 year post-ablation demonstrated that 80.9% of hypo and 84.4% of rhtsh patients had a complete response, defined as complete ablation of all radioiodine uptake in the thyroid bed. In addition, the median serum Tg levels at this follow-up were 0.65 ng/mL and 0.5 ng/mL in the hypo and rhtsh groups respectively. Overall, no significant difference was observed between the groups on measures of successful ablation.

### 3.5 Adverse Events

Some adverse events were associated with both methods of ra preparation. Pacini *et al.* 15 reported mild and transient nausea and fatigue in some patients in both groups. Additionally, some patients in the rhtsh group experienced a loss of taste, and some patients in the hypo group experienced skeletal pain. Barbaro *et al.* 17 reported observing no significant side effects after the administration of rhtsh.

### 3.6 Serum TSH Levels

Mean baseline serum tsh levels in the hypo patients were significantly higher than those in the rhtsh groups 15,17–19. The administration of rhtsh led to substantial increases in serum tsh levels from baseline levels in all the studies that reported this variable. Serum tsh levels are considered sufficient for radioiodine therapy when they reach 25–30 mU/L 6,19. Table III summarizes serum tsh levels.

### 3.7 Iodine Biokinetics

Baseline 24-hour ^131^I uptake, following administration of a tracer dose of ^131^I, was reported in only two papers. In the Pacini *et al.* 18 report, baseline 24-hour ^131^I uptake in the hypo and hypo+rhtsh groups was 5.8% ± 5.7% and 5.4% ± 5.7% respectively and not significantly different. No baseline value was reported for the rhtsh group. Following administration of rhtsh, mean 24-hour ^131^I uptake was 9.4% ± 9.5% in the hypo+rhtsh group, which was significantly higher than the baseline level (*p* < 0.0001), and 2.5% ± 4.3% in the rhtsh group. The value in the rhtsh group was significantly lower than that in the hypo+rhtsh group (*p* < 0.0001). Robbins *et al.* 19 reported median baseline 24-hour ^131^I uptake values that were significantly different between the hypo and rhtsh groups (1.65% and 0.9% respectively, *p* = 0.05), but did not report values following the administration of rhtsh. Barbaro *et al.* 17 evaluated 24-hour ^131^I uptake in only a subset of patients after administration of rhtsh, but found that the hypo and rhtsh groups did not differ significantly (3.30% vs. 2.29%). Only two studies reported post-ablation ^131^I uptake 15,19, and the difference between the hypo and rhtsh groups in each study was not statistically significant. Table IV summarizes 24-hour ^131^I uptake.

Other measures of iodine biokinetics such as mean effective half-life and mean residence time in the remnants are reported only in the paper by Hanscheid *et al.* 20. The effective half-life is a combination of biologic excretion and physical decay; it is the time after which the ^131^I activity drops by 50%. The effective half-life in remnant tissue was significantly shorter (*p* = 0.01) in the hypo group than in the euthyroid group (48 ± 52.6 hours vs. 67.6 ± 48.8 hours). Mean residence times in the hypo and rhtsh groups were 1.4 ± 1.5 hours and 0.9 ± 1.3 hours respectively (not significantly different). Finally, mean dose to the blood was significantly higher in the hypo group than in the rhtsh group (0.167 mGy vs. 0.109 mGy, *p* < 0.0001). Overall, as compared with patients in the hypo group, patients in the rhtsh group received higher doses of radiation to the remnants and a 35% reduction in whole-body radiation dose.

### 3.8 Quality of Life

Baseline qol in the retrieved studies was taken post cancer diagnosis or post surgery and before initiating a protocol for tsh elevation. All the studies reviewed for this outcome demonstrated that qol was worse in the hypo group when compared with either baseline values or with qol in the rhtsh group.

Pacini *et al.* 15 assessed qol using the Billewicz scale and the Medical Outcomes Study Short Form 36 (SF-36). As compared with participants in the rhtsh group, participants in the hypo group scored higher (that is, worse) on 6 of the 14 signs and symptoms of hypothyroidism on the Billewicz scale (*p* < 0.0001). These items included cold intolerance (50% vs. 21%), weight gain (60% vs. 21%), constipation (43% vs. 3%), slow movements (50% vs. 12%), cold skin (47% vs. 12%), and peri-orbital puffiness (50% vs. 0%). In addition, the change from baseline to week 4 in the euthyroid group was significantly different from the change observed in the hypo group for 5 of the 8 health-related qol domains of the SF-36. These domains were physical functioning (*p* < 0.016), role-physical (*p* < 0.018), vitality (*p* < 0.0001), social functioning (*p* < 0.0001), and mental health (*p* < 0.002).

Schroeder *et al.* 12 expressly set out to assess and compare qol in patients undergoing preparation for diagnostic evaluation using tsh withdrawal and rhtsh. Those authors determined that, for all 14 signs and symptoms of hypothyroidism on the Billewicz scale, qol was significantly worse in the hypo group than in the rhtsh group (*p* < 0.001). Likewise, they found that, as compared with patients in the rhtsh group, patients in the hypo group reported significantly lower qol on all 8 health-related qol domains on the SF-36 (*p* < 0.0001) and on the physical and mental composite scores of the SF-36 (*p* < 0.0001 in each case). Ladenson *et al.* 11 also found that qol was significantly worse in the hypo group as compared with the rhtsh group on all signs and symptoms of hypothyroidism on the Billewicz scale (*p* < 0.001). This group of researchers also assessed qol using the Profile of Mood States (poms) and reported that, as compared with the euthyroid group, the hypo group had significantly worse qol on all 6 states measured on the poms (*p* < 0.001). Finally, in 2002, Ladenson 16 compared qol in hypo and rhtsh groups and found that the hypo group had significantly worse qol for 5 of the 6 states assessed on the poms and for the physical composite scores of the SF-36, although probability values were not reported in that article.

### 3.9 Cost Effectiveness

Three studies investigated the economic impact of rhtsh as compared with hormone withdrawal.

Luster *et al.* 21 conducted a retrospective survey. Of patients who were employed in salaried positions outside the home, 62% missed at least 4 days of work (median: 11 days) during their last hypothyroid episode. When outliers were removed from the data, the median number of days missed was still 7.5 (range: 0–70 days). Moreover, 38% of respondents reported taking additional prescription or over-the-counter drugs to relieve hypothyroid symptoms, and 48% reported the use of a primary care physician, a specialist physician, or hospital services because of hypothyroid symptoms. In the economic model presented in the article, the societal cost per diagnostic wbs with preparation by thyroid hormone withdrawal exceeded by 25% or €326 that with preparation by rhtsh 21.

Borget *et al.* 22 compared the frequency, duration, and cost of sick leave for follow-up of thyroid cancer in hormone-withdrawal and rhtsh patients. Active patients in the rhtsh arm were less likely to need sick leave than were those in the hypo arm (11% vs. 33%, *p* = 0.001), and they had shorter sick leaves (3.1 days vs. 11.2 days, *p* = 0.002). Indirect costs owing to absenteeism amounted to €1083 more per person in the hypo group than in the rhtsh group.

Mernagh *et al.* 23 conducted a cost-effectiveness evaluation of rhtsh compared with hormone withdrawal and reported that the benefits of rhtsh are achieved with a small incremental societal cost of €47. That difference equates to an incremental cost-effectiveness ratio of €950 per quality-adjusted life year, which is considered to be very good value for money, considering that new health care interventions are deemed cost-effective if their incremental cost-effectiveness ratio falls below €45,000 23.

## 4. DISCUSSION

There are two fundamental reasons to administer ^131^I following thyroidectomy for differentiated thyroid cancer—namely,

- as adjuvant therapy to destroy the remaining normal thyroid follicular cells to facilitate follow-up and to ease early detection of recurrence, or
- as therapy for disease that proved to be unresectable at surgery.

This review has been concerned only with the first of these indications. Because ablation is directed against residual normal thyroid cells, the specifics of the underlying differentiated malignancy are not relevant to this discussion. Here, we compared only the methods of elevating tsh against the endpoint of successful ablation; we did not address ongoing debates concerning other aspects of thyroid cancer care.

A systematic review of the available evidence on the use of rhtsh compared with thyroid hormone withdrawal in preparation for ra yielded four relevant studies consisting of one rct, two cohort studies, and one retrospective study. Examination of the outcomes of these studies with respect to achieving ablation revealed that the use of rhtsh for ra preparation is not different from thyroid hormone withdrawal. Three of these four studies found similar rates of negative wbs by both methods of preparation 15,17,19. Following surgery and ra, tsh-stimulated serum Tg under either method of preparation is a sensitive indicator of residual thyroid tissue or the presence of cancer (or both). By the criteria of negative wbs and Tg, hypothyroid and euthyroid patients in all four studies had similar rates of successful ablation at 6–12 months post-ablation follow-up 15,17–19.

Serum tsh levels of 25–30 mU/L are considered sufficiently high for radioiodine therapy 6,19. In all the studies reviewed, serum tsh in the hypothyroid patients was sufficient to ensure an adequate uptake of ^131^I. Three studies reported that serum tsh levels after administration of rhtsh were sufficient for the ^131^I uptake needed for ra therapy. Successful ablation was defined by some researchers as a 24-hour ^131^I uptake in the thyroid bed of less than 1% of the administered dose in a follow-up scan 24. Of the two studies reporting that endpoint 15,19, both found 24- hour ^131^I uptake to be less than 1%, with no significant difference between patients prepared by thyroid withdrawal and patients prepared by rhtsh.

In two studies 18,25, a fixed 1110-MBq dose of ^131^I was administered to all patients, but in a more recent study 15, a larger fixed 3700-MBq dose was used. In the Robbins *et al.* 19 study, the ^131^I administered to each individual patient was calculated based on dosimetry. The mean dose in both groups in that study was well above 3700-MBq. The higher dose of ^131^I might result in higher successful ablation rates. Centres continue to differ in the selection of ^131^I dosage, but these differences did not affect our review of the methods employed to elevate tsh.

The report by Hanscheid *et al.* 20 regarding other biokinetic measures in the patients originally studied by Pacini *et al.* 15 found that the mean radiation dose in the remnant was similar in the two groups, but that the mean blood dose, a proxy for whole-body dose, was significantly (30%) lower. That difference is attributable to the impairment of renal function that occurs during hypothyroidism 20.

Investigators continue to explore the limits of safe and effective ^131^I dosage. In a recent rct, Pilli *et al.* 26 compared 1850-MBq and 3700-MBq doses of ^131^I after preparation by rhtsh and demonstrated the same rate of successful ablation (88.9%) in each arm. At the high end of the dose range, Tuttle *et al.* 27 conducted a retrospective analysis of 535 dosimetry studies in hypo patients and concluded that the fixed empiric dosing strategy of 5550–9250 MBq used by many centres frequently results in older patients (70 years of age and older) being exposed to a blood radiation dose of more than 200 cGy, the recommended safety limit. A lower body radiation dose achieved through preparation with rhtsh may lower the risk of radiation-induced second cancers 28, although this effect has not been confirmed in trials. Collectively, the foregoing studies make a compelling argument in favour of rhtsh preparation for ra ablation.

The studies measuring qol 11,12,15,16 consistently reported improved qol in terms of both hypothyroid symptoms and general quality of life for rhtsh as compared with hypo on the Billewicz, SF-36, and poms instruments.

The direct cost of ra by either protocol is identical, except that rhtsh costs $1500 in Canada. The previously cited European studies of cost-effectiveness showed that rhtsh-treated patients had a better qol and, as a result of improved performance capacity, were returning earlier to work and making less use of the health care system. These benefits likely translate to the Canadian environment as well.

Finally, some patients cannot achieve satisfactory levels of endogenous tsh with thyroid hormone withdrawal, and others are contraindicated for hypothyroidism because of other medical disorders. Studies in these populations demonstrated that the use of rhtsh is effective 29,30.

Prospective rcts investigating the therapeutic use of rhtsh are limited in number. Other prospective and retrospective studies have the potential biases inherent in nonrandomized data. However, it is compelling that all the studies retrieved for this review demonstrated the consistent efficacy of rhtsh as a preparation method for ra of thyroid remnants.

The indolence of differentiated thyroid cancers complicates the understanding of long-term outcomes following ablation by whatever protocol. Two recent reports considered the long-term efficacy of ablation therapy given with rhtsh stimulation: one was a follow-up of the Pacini *et al.* series at 3 years 31, and the other was a retrospective review of outcomes in 176 patients at 3–8.5 years later 32. In both cases, no differences were found.

## 5. CONCLUSIONS

Based on current evidence, the efficacy of rhtsh for ra preparation following total or near-total thyroidectomy in patients with papillary or follicular thyroid cancer is equivalent to the traditional method of thyroid hormone withdrawal. In selected patients who cannot tolerate prolonged hypothyroidism or in patients who cannot achieve satisfactory elevation of endogenous tsh by means of thyroid hormone withdrawal, rhtsh may be the only option for radioablation. Recombinant humanized tsh allows patients to remain euthyroid, thereby avoiding the morbidities of hypothyroidism and resulting in better qol than is seen with thyroid hormone withdrawal.

## 6. CONFLICT OF INTEREST DISCLOSURE

JY and RC declare no potential conflicts of interest. AD declares research support from Genzyme Corporation for the development and maintenance of a thyroid cancer database. This author also received honoraria from Genzyme exceeding $5000 annually, all of which have been signed over to the research fund of the Department of Nuclear Medicine at the London Health Sciences Centre.

## Figures and Tables

**TABLE I:** Summary of studies in the literature

Reference	Study type	Comparison	Patients				Thyroid cancer type	Thyroidectomy type
			(n)	Women (n)	Men (n)	Age (years)		
Pacini *et al.,* 2002^18^	Cohort	Hypothyroid	50	36	14	17–75	Papillary or follicular	Total or near-total
		Hypothyroid + rhtsh	42	30	12			
		rhtsh	70	53	17			
Robbins *et al.,* 2002^19^	Retrospective	Hypothyroid	42	17	25	42.2±17.5	Papillary	Total or near-total
		rhtsh	45	27	18	49.9±13.3^a^		
Barbaro *et al.,* 2006^17^	Cohort	Hypothyroid	41	26	15	19–71	Papillary or minimally invasive follicular	Total
		rhtsh	52	35	17			
Pacini *et al.,* 2006^15^	Randomized controlled trial	Hypothyroid	30	24	6	20–68	Papillary or follicular	Total or near-total
		rhtsh	33	26	7			

rhtsh = recombinant humanized thyroid-stimulating hormone.

a *p*<0.05.

**TABLE II:** Rates of successful ablation and levels of serum thyroglobulin (Tg), by study

Reference	Comparison	^131^I Dose	Successful ablation (negative wbs) (%)	Serum Tg < 1 ng/mL at 6–12 months’ follow-up (%)
Pacini *et al.,* 2002^18^	Hypothyroid	1110 MBq	84.0 (*p*<0.0001)^a^	83.3^b^
	Hypothyroid + rhtsh		78.5 (*p*<0.01)^a^	84.8^b^
	rhtsh		54.0	86.8^b^
Robbins *et al.,* 2002^19^	Hypothyroid	Based on dosimetry^c^	80.9	0.65 ng/mL^d^
	rhtsh		84.4	0.50 ng/mL^d^
Barbaro *et al.*, 2006^17^	Hypothyroid	1110 MBq	75.6	78.0
	rhtsh		76.9	86.5
Pacini *et al.,* 2006^15^	Hypothyroid	3700 MBq	86.0	86.0
	rhtsh		75.0	83.0

a Compared with rhtsh alone.

b Based on patients whose ablation was successful.

c Mean ± standard deviation dose was 4769.3 ± 2738 MBq and 4084.8 ± 2405 MBq for the hypothyroid and rhtsh groups respectively.

d Median Tg level.

wbs = whole-body scan; rhtsh = recombinant humanized thyroid-stimulating hormone.

**TABLE III:** Serum levels of thyroid-stimulating hormone (tsh)

Reference	Comparison	Mean serum tsh ± standard deviation (mU/L)	
		Baseline	24 Hours after rhtsh
Pacini *et al.,* 2002^18^	Hypothyroid	63.2±19.6^a^	na
	Hypothyroid + rhtsh	71.0±35.9^a^	281±97.0^a^ (*p*<0.0001)
	rhtsh	1.30±2.5^a^ (*p*<0.0001)	126±44.8^a^ (*p*<0.0001)
Robbins *et al.,* 2002^19^	Hypothyroid	97.5±50	na
	rhtsh	6.0±9.5	105.1±45.4
Barbaro *et al.,* 2006^17^	Hypothyroid	50±3	na
	rhtsh	0.04–0.35^b^	126±10
Pacini *et al.,* 2006^15^	Hypothyroid	83.0±51	na
	rhtsh	1.1±1.3	nr

a Pacini *et al.* (2002)^18^ report serum tsh levels in microunits per millilitre, which is equivalent to milliunits per litre.

b Range reported only.

rhtsh = recombinant humanized tsh; na = not applicable; nr = not reported.

**TABLE IV:** Iodine biokinetics in remnants: 24-hour ^131^I uptake (mean ± standard deviation)

Reference	Comparison	Before rhtsh	24-Hour ^131^I uptake Hypothyroid or after rhtsh	After ablation
Pacini *et al.,* 2002^18^	Hypothyroid	5.8±5.7	na	nr
	Hypothyroid + rhtsh	5.4±5.7	9.4±9.5 (*p*<0.0001)^a^	nr
	rhtsh	nr	2.5±4.3 (*p*<0.0001)^b^	nr
Robbins *et al.,* 2002^19^	Hypothyroid	1.65^c^	na	0.25^c^
	rhtsh	0.9^c^ (*p*=0.05)	nr	0.17^c^
Barbaro *et al.,* 2006^17^	Hypothyroid	nr	3.30±0.7^d^	nr
	rhtsh	nr	2.29±0.45^d^	nr
Pacini *et al.,* 2006^15^	Hypothyroid	nr	na	0.9±1.1^e^
	rhtsh	nr	nr	0.5±0.7^e^

a Comparison within the hypothyroid + rhtsh group, before and after rhtsh.

b Comparison between the hypothyroid + rhtsh group and the rhtsh group after rhtsh.

c Median value.

d Evaluated on a subset of patients only.

e 48-Hour ^131^I uptake.

rhtsh = recombinant humanized tsh; nr = not reported; na = not applicable.
